# Supplementary material for: Utilization of non-pneumatic anti-shock garment and associated factors for postpartum hemorrhage management among obstetric care providers in public health facilities of southern Ethiopia, 2020
Source: PLoS One. 2021 Oct 28;16(10):e0258784. doi: 10.1371/journal.pone.0258784 (PMC8553034; doi:10.1371/journal.pone.0258784)
Supplement: S1 File — (DOCX) [file pone.0258784.s001.docx]

## **Annex II: English Version Participant Information Sheet and Informed**

## **Voluntary Consent for health professionals**

My name is ___________________. I am working as a data collector for the study being conducted in this hospital by Mrs. Yordanos Gizachew who is a lecturer at Arba Minch University. I kindly request you to lend me your attention to explain you about the study and your institution being selected as the study setting.

**The study title:** Utilization of health professionals on the Use of Non-pneumatic Anti-Shock Garment in Control of Post-partum Hemorrhage and associated factors in public health institutions of south west , Ethiopia

**Purpose of study:** The findings of this study can be important for the healthcare providers working in maternity unit to identify the factors to scale-up NASG utilization. Besides, the study will be used for the hospital managers to prepare a written guidelines and protocols for the management complications from PPH using NASG. It can also provide important information to policymakers, and program managers to address such issues in the future

**Procedure and duration:** Data will be collected from health professionals working in maternity units. The interview will take about 40 minutes, so I kindly request you to give permission to collect data pertinent to this study.

**Risks and benefits:** The risk of participating in this study is very minimal, but only taking few minutes from the health professionals time. There would not be any direct payment for participating in this study. But the findings from this research may reveal important information for the hospital and local health planners.

**Confidentiality:** The information that we will be provided will be kept confidential. There will be no information that will identify the participants in particular. The findings of the study will be general for the study community and will not reflect anything particular of individual persons. The questionnaire will be coded to exclude showing names. No reference will be made in oral or written reports that could link participants to the research.

**Rights:** Participation for this study is fully voluntary. The participants have the right to declare to participate or not in this study. If they decide to participate, they have the right to withdraw from the study at any time and this will not label them for any loss of benefits which they otherwise are entitled. They do not have to answer any question that they do not want to answer.

**Contact address:** If you have any questions about the study, the procedure or anything else related to the study, please contact through the following address:

Institutional Ethics Board (IRB), College of Medicine and Health Sciences, Arba Minch University: Office phone: _______________ P.O.BOX: 21, Arba Minch.

**Declaration of informed voluntary consent:** I have read the institution information sheet. I have clearly understood the purpose of the research, the procedures, the risks and benefits, issues of confidentiality, the rights of participants and the contact address for any queries. I have been given the opportunity to ask questions for things that may have been unclear. I was informed that participants have the right to withdraw from the study at any time or not to answer any question that they do not want. Therefore, I declare my voluntary consent to participate in this study with my initials (signature).

Name and Signature of Head of the Hospital: __________________________

Name and Signature of Data Collector: ______________________________

**Annex III: English Version Questionnaire**

| Name of the institution : ________________________________ | | | | | | | | |
| --- | --- | --- | --- | --- | --- | --- | --- | --- |
| Code number of the questionnaire: ______________________________ | | | | | | | | |
| Interviewer’s name & signature: | | Name:___________________ | | | | Signature_________ | | |
| Supervisor’s name & signature: | | Name :__________________ | | | | Signature__________ | | |
| **S/NO** | **Question** | | **Response** | | | | **Skip** | |
| **Part I: Socio-demographic Characteristics** | | | | | | | | |
|  | How old are you? ( in completed years) | | **______________** | | | |  | |
|  | What is your religion? | | 1. Protestant 2. Orthodox 3. Muslim 4. Catholic 5. Other(specify)________ | | | |  | |
|  | What is you Gender ? | | 1. Female 2. Male | | | |  | |
|  | What is your marital status? | | 1. Single 2. Married 3. Divorced 4. Widowed 5. Married but living apart | | | |  | |
|  | Where is your residency ? | | 1. Urban 2. Rural | | | |  | |
|  | What is your qualification ? | | 1. Diploma nurse 2. Diploma midwifery 3. Degree nurse 4. Degree midwifery 5. IESO 6. Masters midwifery 7. Masters nurse 8. General physician | | | |  | |
|  | Year of experience | | ____________ | | | |  | |
| **Part II: facility related Characteristics** | | | | | | | | |
| 201 | Type of facility | | 1. District hospital 2. General hospital 3. Health center | | | |  | |
| 202 | Have you had Trainings about NASG in your hospital ? | | 1. Yes 2. No | | | |  | |
| 203 | Are their protocols about NASG management in your hospital? | | 1. Yes 2. No | | | |  | |
| 204 | Are there wall charts displayed in your hospital about NASG? | | 1. Yes 2. No | | | |  | |
| 205 | Have you had trainings about misoprostol in you hospital? | | 1. Yes 2. No | | | |  | |
| 206 | Are their protocols about misoprostol management in your hospital? | | 1. Yes 2. No | | | |  | |
| 207 | Are there wall charts displayed in your hospital about misoprotol ? | | 1. Yes 2. No | | | |  | |
| 208 | Is there a trend of staff motivation/incentive in your hospital | | 1. Yes 2. No | | | |  | |
| 209 | If yes to question 208 , what is the form of motivation or incentive | | __________________ | | | |  | |
| **Part III: Contextual factors** | | | | | | | | |
| 301 | Where is your unit/ ward in a hospital? | | 1. ANC 2. FP 3. Labour and delivery 4. Gyn OPD 5. Post-natal | | | |  | |
| 302 | How many duties do you have in a month? | | _________________ | | | |  | |
| 303 | With whom are you placed while you are on duty? | | 1. With coworkers 2. Alone | | | |  | |
|  | Please categorize the flow of obstetric emergency in your seating? | | 1. Frequent 2. Intermediate 3. Rare | | | |  | |
| 305 | Do you have experienced staff in your unit/ward? | | 1. Yes 2. No | | | |  | |
| **Part IV: Knowledge of respondent on NASG** | | | | | | | | |
|  | Have you heard of the non-pneumatic antishock garment | | 1. Yes 2. No | | | |  | |
|  | Have you ever seen the NASG | | 1. Yes 2. No | | | |  | |
|  | Where did you first hear it  (multiple answers are possible) | | 1. Hospital /health center 2. School 3. Seminar 4. Internet 5. Printed material 6. Media 7. Friends and colleague | | | |  | |
|  | What is the NASG like? | | 1. Bottom half of a suit 2. A gown 3. A trouser | | | |  | |
|  | Do you know what the NASG is used for | | 1. Yes 2. No | | | |  | |
|  | If yes please state the use | | _________________________ | | | |  | |
|  | What is it made of | | 1. Velcro 2. neoprene | | | |  | |
|  | The Non-Pneumatic Antishock Garment is a light weight washable and inexpensive garment. | | 1. Yes 2. No | | | |  | |
|  | The Non-Pneumatic Antishock Garment is a device for resuscitating patient from shock | | 1. Yes 2. No | | | |  | |
|  | How many segements does the NASG have? | | 1. Four 2. Six 3. Nine 4. Five | | | |  | |
|  | The NASG work by forcing blood from the lower part of the body to vital organs | | 1. Yes 2. No | | | |  | |
|  | The NASG can also be used when a woman is already in shock | | 1. Yes 2. No | | | |  | |
|  | A patient with the Non-Pneumatic Antishock Garment (NASG) on can undergo any investigation to find out the cause of the haemorrhage. | | 1. Yes 2. No | | | |  | |
|  | When applied on a patient, the Non-Pneumatic Antishock Garment applies a circumferential pressure. | | 1. Yes 2. No | | | |  | |
|  | Which procedure can be performed on a women on NASG?  (multiple answers are possible) | | 1. IV line 2. Vaginal surgery 3. Abdominal surgery 4. Transport to other facility 5. All of the above | | | |  | |
|  | NASG Saves time, energy and life | | 1. Yes 2. No | | | |  | |
|  | NASG is Inexpensive, comfortable, durable and easy to maintain | | 1. Yes 2. No | | | |  | |
|  | NASG Reduces further blood loss | | 1. Yes 2. No | | | |  | |
|  | Can be on the patients for several hours without adverse consequences | | 1. Yes 2. No | | | |  | |
|  | Prevents maternal morbidity and mortality | | 1. Yes 2. No | | | |  | |
|  | When should the NASG be removed?  (multiple answers are possible) | | 1. After stabilizing for 2hrs 2. When the hg is 7g/ dl or more and hematocrit of about 20% 3. Pulse rate less than 100 bpm 4. Diastolic bp 90mmhg or more 5. When the women is awake /stable 6. Bleeding <50ml/hr | | | |  | |
|  | When applying the NASG, from which segment do we start? | | 1. Abdominal segment 2. Lower or ankle segment | | | |  | |
|  | When removing the NASG, from which segment do we start? | | 1. Abdominal segment 2. Lower or ankle segment | | | |  | |
|  | At what time interval do we need to remove each segment? | | 1. Each successively 2. 15 minutes apart 3. 1hr apart | | | |  | |
|  | If the woman experiences difficulty breathing with the NASG, the provider may adjust the abdominal panel. | | 1. Yes 2. No | | | |  | |
|  | How long the NASG can/should be used on a given patient? | | 1. For two hours 2. For 48 hours 3. Can be applied a for hours or days until the bleeding has been arrested | | | |  | |
|  | Contra-indication to the use of NASG.  (multiple answers are possible) | | 1. Viable fetus 2. Dyspnea 3. Mitral stenosis 4. Congestive heart failure 5. Pulmonary hypertension 6. Bleeding above the level of diaphragm 7. All of the above | | | |  | |
|  | How many times can the NASG be disinfected and washed | | 1. At least 30 times. 2. It is only used once 3. 10 times | | | |  | |
|  | How can you ensure that the NASG is free of the HIV and hepatitis virus?  (multiple answers are possible) | | 1. Put it out in the sun to dry 2. Decontaminate the garment with a 0.05% chlorine solution 3. Wash the garment with soap and water or in a washing machine 4. All of the above | | | |  | |
|  | How do you decontaminate the NASG after use? | | 1. By 0.05% bleach solution, 2. By 0.5 % bleach solution | | | |  | |
| **Section 5: Attitude of respondents towards non pneumatic antishock garment** | | | | | | | | |
|  | The use of anti-shock garment (NASG) is unnecessary especially in center where there is facility for blood transfusion | | Strongly agree | Agree | Undecided | | Disagree | Strongly disagree |
|  | There is no need of the garment (NASG), since it is not readily available | |  |  |  | |  |  |
|  | The garment (NASG ) is expensive, therefore not affordable | |  |  |  | |  |  |
|  | The garment (NASG) application and removal requires a lot of procedures that takes time. | |  |  |  | |  |  |
|  | The garment (NASG ) can transmit HIV to patients; hence it is not advisable to be used in a hospital setting | |  |  |  | |  |  |
|  | Anti-shock garment (NASG ) is only beneficial to people in the rural areas/primary care settings | |  |  |  | |  |  |
|  | The garment (NASG) is only meant to be utilized by doctors | |  |  |  | |  |  |
|  | Anti-shock garment (NASG ) is ineffective in patients with cervical lacerations | |  |  |  | |  |  |

|  | **Section 6 :Utilization of respondent** | | |
| --- | --- | --- | --- |
|  | Have you ever managed a patient with PPH? | 1. Yes 2. No |  |
|  | Have you ever administered anti-shock garment to any patient? | 1. Yes 2. No | If no go to  709 |
|  | If yes, how many often? | 1. Rarely 2. Sometimes 3. Often 4. Always |  |
|  | Have you been trained in the use of Anti-shock garment | 1. Yes 2. No |  |
|  | When was the last time you used anti-shock garment? | 1. Less than 1 month ago 2. Between 1 month – <3 months ago 3. Between 3 months – 6 months ago 4. More than 6 months ago |  |
|  | What was the clinical condition of the last patient you used anti-shock garment for? | 1. Obstructed labour 2. Post-partum haemorrhage 3. Eclampsia 4. Hypovolaemic shock 5. Others________ |  |
|  | What was the outcome of that patient? | 1. Recovery 2. Referral 3. Death |  |
|  | If No to question 702 , why not? | 1. Didn’t know it was available 2. No patient needed it 3. Don’t know how to use it 4. Others_______ |  |
|  | NASG is part of your health care protocol for PPH before referral. | 1. Yes 2. No |  |
|  | If yes, was it effective | 1. Yes 2. No |  |
|  | Do you use NASG every time there is PPH | 1. Yes 2. No |  |
|  | If no, when do you use it | 1. Severe PPH 2. Shock 3. When other method fail |  |
|  | Do you use it, when the need arise in your health center | 1. Yes 2. No |  |
|  | If no why | 1. It is difficult to assemble 2. It is not available 3. I do not know much about it |  |
|  | If you know how to use it will you use it | 1. Yes 2. No |  |
|  | If it is available will you use it | 1. Yes 2. No |  |
|  | Have you ever administered misoprostol to any patient? | 1. Yes 2. No 3. If no, go to question 721. |  |
|  | If yes, how often? | 1. Rarely 2. Sometimes 3. Often 4. Always |  |
|  | When was the last time you used misoprotol? | 1. Less than 1 month ago 2. Between 1 month – <3 months ago 3. Between 3 months – 6 months ago 4. More than 6 months ago |  |
|  | 1. What was the clinical condition of the last patient you gave misoprostol? | 1. Obstructed labour 2. Post-partum haemorrhage 3. Eclampsia 4. Hypovolaemic shock 5. Others_____________________ |  |
|  | What was the outcome of that patient? | 1. Recovery 2. Referral 3. Death |  |
|  | If no to question 716 , Why not? | 1. Didn’t know it was available 2. No patient needed it 3. Don’t know how to use it 4. Others_______________ |  |
