# Supplementary material for: Utilization of non-pneumatic anti-shock garment and associated factors for postpartum hemorrhage management among obstetric care providers in public health facilities of southern Ethiopia, 2020
Source: PLoS One. 2021 Oct 28;16(10):e0258784. doi: 10.1371/journal.pone.0258784 (PMC8553034; doi:10.1371/journal.pone.0258784)
Supplement: Attachment — Submitted filename: Response to Reviewers.doc [file pone.0258784.s003.doc]

**Title:** Utilization of non-pneumatic anti-shock garment and associated factors for postpartum hemorrhage management among obstetric care providers in public health facilities of southern Ethiopia, 2020

**Authors:**

Yordanos Gizachew Yeshitila (yordanos.gizachew@yahoo.com)

Agegnehu Bante (agegnehubante@gmail.com)

Zeleke Aschalew (zelekeaschalew@gmail.com)

Bezawit Afework (bezawitafework2010@gmail.com)

Selamawit Gebeyehu ([emugebe.sg@gmail.com](mailto:emugebe.sg@gmail.com))

**Responses compiled by**: Yordanos Gizachew Yeshitila

On behalf of the authors

| **Responses to editor’s comment**  **Editor’s comments, suggestions and question are considered and carefully revised the manuscript as per the suggestions and comments. Thank you for the feedback. We have addressed each comment below and within the revised manuscript (tracked changes and clean version).**  Sincerely,  Yordanos Gizachew Yeshitila  on behalf of the authors | |
| --- | --- |
| Please include the following items when submitting your revised manuscript:   - A rebuttal letter that responds to each point raised by the academic editor and reviewer(s). You should upload this letter as a separate file labeled 'Response to Reviewers'. - A marked-up copy of your manuscript that highlights changes made to the original version. You should upload this as a separate file labeled 'Revised Manuscript with Track Changes'. - An unmarked version of your revised paper without tracked changes. You should upload this as a separate file labeled 'Manuscript' | - Thank you dear editor, we have included all the three requested items |
| When submitting your revision, we need you to address these additional requirements.  1. Please ensure that your manuscript meets PLOS ONE's style requirements, including those for file naming. The PLOS ONE style templates can be found at  https://journals.plos.org/plosone/s/file?id=wjVg/PLOSOne_formatting_sample_main_body.pdf and  <https://journals.plos.org/plosone/s/file?id=ba62/PLOSOne_formatting_sample_title_authors_affiliations.pdf> | Dear editor, we have gone through the manuscript, and revised it according to the PLOS ONE's style requirement |
| We note that the grant information you provided in the ‘Funding Information’ and ‘Financial Disclosure’ sections do not match. When you resubmit, please ensure that you provide the correct grant numbers for the awards you received for your study in the ‘Funding Information’ section. | Dear editor, we apologize for the inconsistency, we provided correct grant numbers for the awards we received for our study in the ‘Funding Information’ section |
| In your Data Availability statement, you have not specified where the minimal data set underlying the results described in your manuscript can be found. PLOS defines a study's minimal data set as the underlying data used to reach the conclusions drawn in the manuscript and any additional data required to replicate the reported study findings in their entirety. All PLOS journals require that the minimal data set be made fully available. For more information about our data policy, please see http://journals.plos.org/plosone/s/data-availability. | Dear editor, we have corrected the data availability statement as per the journal requirement |
| Upon re-submitting your revised manuscript, please upload your study’s minimal underlying data set as either Supporting Information files or to a stable, public repository and include the relevant URLs, DOIs, or accession numbers within your revised cover letter. For a list of acceptable repositories, please see http://journals.plos.org/plosone/s/data-availability#loc-recommended-repositories. Any potentially identifying patient information must be fully anonymized. | Dear editor, we have uploaded study’s minimal underlying data set as Supporting Information files |
| Please include captions for your Supporting Information files at the end of your manuscript, and update any in-text citations to match accordingly. Please see our Supporting Information guidelines for more information: http://journals.plos.org/plosone/s/supporting-information. | Dear editor, we have included captions for our Supporting Information files at the end of our manuscript, and updated any in-text citations to match accordingly |
| I appreciate the authors' work on this paper and the findings are interesting. As noted by the reviewers, a number of revisions are needed as well as editing for language and grammar. Please also make the data available to ensure compliance with PLoS One's open data policy. | Dear editor, thank you so much. We have gone through each and every concern and comments raised, and responded accordingly. We have also exhaustively worked on the editing for language and grammar. |
| **Responses to the Reviewers' comment and question**  **Reviewer’s comments and suggestions are considered and carefully revised the manuscript as per the suggestions and comments. I have addressed the requests and concerns raised by both the reviewers. I am very thankful to the potential reviewers for suggestions and comments, which substantially improved the manuscript** | |
| **Reviewer 1 comment and question** | **Responses Reviewer 1** |
| The aims of this study are valuable, and the authors’ recommendations for increasing the use of NASG by targeting primary health centers are a valuable message. However there are some issues in the reporting of the results and conclusions that should be clarified to reflect the data. In particular, the conclusion should recognize that the Ethiopian government and CHAI have achieved a significant victory if within a random sample of providers, nearly half are using the NASG for management of hypovolemic shock and PPH. Several other countries are working towards this goal but are not close to this achievement. Attention should also be paid to the interpretation of the scaled attitude data. The authors should be attentive to reporting this data in an objective manner. Otherwise, the authors’ conclusions appear sound and provide important feedback about the introduction of the NASG in Ethiopia | Dear reviewer, thank you for your insights and thank you so much for meticulous observation and comments which significantly improved the manuscript.  *We have considered and incorporated your suggestion on the interpretation of the scaled attitude data. |
| The data for this study is indicated as being fully available according to PLOS One’s guidelines. However some data has been simplified for analysis and is not available in its original form within the supplemental material. I have provided suggestions for how this could be corrected within the manuscript in comment #9. | Dear reviewer, we have provided the data as per your request.  (**Page 11-12)** |
| **Language:** a. Please correct the spelling of “professionals” in the abstract and throughout the text. b. Please correct “settlement” to “establishment” in the abstract and throughout the text. c. “nasg” should be capitalized NASG as an acronym throughout the text. d. Authors should check spelling and capitalization throughout the text. | Dear reviewer, we truly appreciate your suggestions and comments. We have incorporated each of them in the revised manuscript. The changes we made are presented on the revised manuscript with track change. |
| Line 40 “A significant proportion of obstetric care providers do not use non pneumatic antishock garment for preventing complications from postpartum hemorrhage.”  This is a subjective and possibly misleading statement, as the authors do not give a reference point for comparison. It would be more accurate to say “roughly half of providers are using the NASG,” and then go on to describe the reasons the authors would recommend that actions be taken to increase this proportion | Dear reviewer, we have corrected the sentences as per your suggestion.  **Page 2, line 43-44 and page21, line 365-366** |
| Line 81. Add period: “excessive bleeding. With the introduction…” | Dear reviewer, we have made the correction. **Page 3, line 80** |
| Lines 103-105 “Most the studies conducted before used a small sample size (60-112) [19-22] and in developed countries where maternal mortality is less than 64/100 000 [23-25]."  If the authors are referring to implementation studies (studies examining utilization), both parts of this statement are not accurate. Please refer to Mbaruku study was conducted with a sample size of 1,713 women in Tanzania, published in 2018.  https://pubmed.ncbi.nlm.nih.gov/30340602/ | Dear reviewer, thank you for thorough review of our work.  The first part of the paragraph was intended to show those studies conducted on professionals knowledge and use of NASG, the references listed, and the sample size they used (in brackets) were Faiza et al (60), Kombo et al (100), Kolade et al (110), and Onasoga et al (112), however, for the second part of the statement as you have provided us with the references there are studies conducted in developing countries, where we were trying to show the studies from developed countries. So we removed the second part of the statement.  **(Page4, line 98-99)** |
| Line 234-235 Regarding the use of the NASG on patients with a viable fetus. This is perhaps not worth mentioning in this paper as this issue is not being explored in-depth. Conclusive studies have not been done to investigate safety of the NASG with a viable fetus, and the NASG can be considered for use when “there is no other way to save the mother’s life and both mother and fetus will die.” (https://www.nqocncop.org/forum/welcome-to-the-forum/complementary-role-of-non-pneumatic-anti-shock-garment-nasg-in-management-hemorrhagic-shock-in-obstetrics) | Dear reviewer, thank you again for your meticulous observation. It has been stated there is no absolute contraindication for use of NASG for PPH, However, regarding the use of NASG, as an indication, it is stated on pathfinder’s guideline that (page 87) “*The NASG could be used to manage any condition where there is severe bleeding below the diaphragm. Our studies have documented use with all forms of obstetric hemorrhage, as long as the fetus is not viable in utero”*  We would appreciate if you take look at the guideline (<https://www.pathfinder.org/publications/prevention-recognition-management-of-postpartum-hemorrhage-trainers-guide/> |
| Line 238: Table 3  For the knowledge questions, the authors could consider indicating all the correct responses for the readers who may not have this information. | Dear reviewer, we have indicated the correct responses on the table with symbol and provided description for the symbols underneath the table. |
| Line 248 “Over all, 245 (59.5%) of the respondts had unfavourbale attitude towards non neumatic antishock garment.”  This statement appears to be attempting to summarize the results of the 8 scaled attitude statements, and does not appear to be a sound conclusion. It's not clear how the number 245 was derived. If I understand the author’s interpretation, this statistic counts neutral responses as “unfavorable” rather than neutral. Please see the next comment for feedback on the interpretation of the Likert scale responses. This statement should be removed and replaced with information that is firmly rooted in the data, however I am not able to provide more guidance as not enough information about the attitude responses has been made available in the manuscript. | Dear reviewer, we are grateful for the comments and lesson on this specific issue, As you have emphasized, the neutral or undecided attitude of the professionals is another core aspect for future programmers and interventional projects on the area regarding NASG utilization.  *We have provided data on the neutral or undecided attitude aspect of the professionals **(Page 11-12)**  *The whole analysis was changed and interpreted again accounting for the neutral attitude  **(Page 16-17)**  ***** We have also included under discussion part **(Page9, line 311-325** |
| Table 4:  Attitudes  Reporting Likert scale data can pose a challenge. The 5 point Likert scale used here allowed respondents to report a neutral or undecided response. However the authors have chosen to collapse the 5 possible responses into a binary variable, “favorable” or “unfavorable”. Collapsing 5 responses without differentiation is problematic. Characterizing neutral responses as negative can mislead the reader and impacts the authors’ interpretation.  “Undecided” responses are important within this context as they indicate the number of respondents who may feel insufficiently informed on the NASG. A respondent who feels uninformed about the NASG should be considered substantively different from a respondent who feels informed and has formed a negative attitude. The neutral response is in line with the “I don’t know” categories in Table 3. It is relevant and important for those who are programming activities on the NASG to know the scope of uniformed staff within the health facilities, and this information supports the authors’ conclusion that further training is required. This data should be clearly presented to the readers. The authors should add at least 1 column to this table to show the neutral responses, or alter this table to include 5 columns, to reflect all possible responses to gain the full benefit of the Likert scale. Including the “strongly agrees” and “strongly disagrees” would add value to the authors ‘ work. This data can be represented in a table or a graph displaying all percentages. An example of clear representation of 5-point responses can be found in this article:  Haffer H, Schömig F, Rickert M, Randau T, Raschke M, Wirtz D, Pumberger M, Perka C. Impact of the COVID-19 Pandemic on Orthopaedic and Trauma Surgery in University Hospitals in Germany: Results of a Nationwide Survey. J Bone Joint Surg Am. 2020 Jul 15;102(14):e78. doi: 10.2106/JBJS.20.00756. PMID: 32675666; PMCID: PMC7431148.  In addition, I would recommend that “favorable” be changed to “disagree” and “unfavorable” be changed to “Agree” which are the standard convention in English.  These changes should ensure that readers do not misinterpret the important results in this table. These changes should also be reflected in Table 6, and discussed in the results. | Thank you dear reviewer,  We have reviewed the article you suggested (<https://journals.lww.com/jbjsjournal/Fulltext/2020/07150/Impact_of_the_COVID_19_Pandemic_on_Orthopaedic_and.6.aspx>) and  We have provided data on the neutral or undecided attitude aspect of the professionals **(Page 11-12)**   - We have changed that “favorable” be changed to “agree” and “unfavorable” be changed to “disagree” |
| Line 284 “substantiate” is not correct in this context. Sentence could read “The NASG has been proven effective as a tool to stabilize….” | Dear reviewer, we have revised it as per your suggestion, **(Page 17, line 277)** |
| Line 285 Replace “absolute” with “definitive.” | Dear reviewer, we have revised it as per your suggestion **Page 17, line 278)** |
| Line 311 “inception” The authors’ intention with this word is not clear. Possibly “initial” or “new hire trainings”? | Dear reviewer, the inception training were meant to be new hire training, as per your suggestion, we have corrected for clarity (**Page 18, line 304)** |
| Line 365 “A significant proportion of obstetric care providers do not use non pneumatic antishock garment for preventing complications from postpartum hemorrhage.” Same correction recommended as line 40. | Dear reviewer, We have made the correction |
| **Reviewer 2 comment and question** | **Responses Reviewer 2**  Dear reviewer, thank you for your insights and thank you so much for meticulous observation and comments which significantly improved the manuscript. |
| Please correct the grammar and some spelling errors in the manuscript. | Dear reviewer, thank you. We have gone through the manuscript and made all the possible corrections. (changes can be found on the tracked version) |
| Please mention whether all the obstetric care workers in Southern Ethiopia have previously been offered training on how the NASG is used. | Dear reviewer, thank you. We have included obstetric care providers in health facilities where NASG is available, however weather they received training or not was one of the variable we assessed on table 2 (Page 10-11). |
| Also mention whether refresher trainings are available. | Dear reviewer, same as to the above concern, we have included obstetric care providers in health facilities where NASG is available, however weather they received training or not was one of the variable we assessed on table 2 **(Page 10-11).** |
| Also indicate whether the NASG is actually available at the institutions where the study was done. | Dear reviewer, thank you. We have included obstetric care providers in health facilities where NASG is available, we have incorporated this under the method section, under subsection Study design and population **(Page 6 line 130-132)** |
| On page 13 please indicate the type of random sampling that was used to select the institutions that participated in the study. | Dear reviewer, thank you. We have indicated on **page 6, line 143** |
| The title of Table 1 gives the impression that these are the socio-demographic characteristics of all obstetric care workers in the region. Better to title the table to indicate that these are the socio-demographic characteristics of only the obstetric care workers who participated in the study | Dear reviewer, we truly appreciate your suggestion, we have revised the title as per your suggestion. **(Page 9, line 202-203)** |
| On Table 2 under ''Professions'' what does ''Emergency surgery'' refer to? Is it referring to general surgeons? On page 18 (lines 229 to 231) what denominator was used to determine the proportion of respondents that knew that the NASG is made up of six parts? | Dear reviewer, we apologize for using the short version of the title. The profession is Integrated Emergency Obstetric Surgery, not general surgeons. We would appreciate if you visit the link for better clarification (<https://ethiopia.unfpa.org/en/news/msc-programme-integrated-emergency-obstetric-surgery-launched> |
| On page 18 (lines 229 to 231) what denominator was used to determine the proportion of respondents that knew that the NASG is made up of six parts? | Dear reviewer, we have used the denominator of 338 (those who heard about NASG) we have included the denominator for each question. |
| On Table 3 (page 19) was the third row from the bottom meant to say ''Transport to other facilities''? | Dear reviewer, thank you. Yes, it is mean to say ''Transport to other facilities''?, we have modified on the table |
| The type of questions in Table 4 and Table 5 ideally should not be asked of someone who says that they have never heard of the NASG. The denominators should exclude those who say that they have never heard of the NASG. | Dear reviewer, we are so much grateful for the suggestion, which we believe clear confusion for future readers. We have included the denominator for each question. |
| **Additional corrections made by the author** | |
| *We have exhaustively reviewed the manuscript as suggested by the reviewers and modified accordingly and added some modifications too (Please refer to the track change version of the manuscript)  *We have included two supporting information’s one for responding inquiries of the reviewers **(manuscript with track change),** and the second for professional edits and exhaustive review of the manuscript (**supporting information2)** |  |
