# Supplementary material for: Utilization of non-pneumatic anti-shock garment and associated factors for postpartum hemorrhage management among obstetric care providers in public health facilities of southern Ethiopia, 2020
Source: PLoS One. 2021 Oct 28;16(10):e0258784. doi: 10.1371/journal.pone.0258784 (PMC8553034; doi:10.1371/journal.pone.0258784)
Supplement: Attachment — Submitted filename: Response to Reviewers.doc [file pone.0258784.s004.doc]

**Title:** Utilization of non-pneumatic anti-shock garment and associated factors for postpartum hemorrhage management among obstetric care providers in public health facilities of southern Ethiopia, 2020

**Authors:**

Yordanos Gizachew Yeshitila (yordanos.gizachew@yahoo.com)

Agegnehu Bante (agegnehubante@gmail.com)

Zeleke Aschalew (zelekeaschalew@gmail.com)

Bezawit Afework (bezawitafework2010@gmail.com)

Selamawit Gebeyehu ([emugebe.sg@gmail.com](mailto:emugebe.sg@gmail.com))

**Responses compiled by**: Yordanos Gizachew Yeshitila

On behalf of the authors

| **Responses to editor’s comment**  **Dear editor, Thank you for the feedback. We have addressed comments and suggestions below and within the revised manuscript (tracked changes and clean version).**  Sincerely,  Yordanos Gizachew Yeshitila  on behalf of the authors | |
| --- | --- |
| Please include the following items when submitting your revised manuscript:   - A rebuttal letter that responds to each point raised by the academic editor and reviewer(s). You should upload this letter as a separate file labeled 'Response to Reviewers'. - A marked-up copy of your manuscript that highlights changes made to the original version. You should upload this as a separate file labeled 'Revised Manuscript with Track Changes'. - An unmarked version of your revised paper without tracked changes. You should upload this as a separate file labeled 'Manuscript' | - Thank you dear editor, we have included all the three requested items |
| Please review your reference list to ensure that it is complete and correct. If you have cited papers that have been retracted, please include the rationale for doing so in the manuscript text, or remove these references and replace them with relevant current references. Any changes to the reference list should be mentioned in the rebuttal letter that accompanies your revised manuscript. If you need to cite a retracted article, indicate the article’s retracted status in the References list and also include a citation and full reference for the retraction notice. | Dear editor, thank you, we have gone tough the reference and made correction, we have showed the changes we made with highlight on the track version of the manuscript.  We have also mentioned the changes we made to reference based on the suggestion from one of the reviewer on the cover letter. |
| My thanks to the authors for undertaking this revision, which have improved the manuscript. In addition to the remaining critiques from the reviewers, please ensure the de-identified data set is included with the next revision, as required by the journal. | Dear editor, we are grateful for the feedbacks. We have uploaded the de-identified data set which is used for the study. |
| **Responses to the Reviewers' comment and question**  **Reviewer’s comments and suggestions are carefully considered and revised the manuscript as per the suggestions and comments. I have incorporated the suggestions and comments raised by both the reviewers. I am very thankful to the potential reviewers for suggestions and comments, which substantially improved the manuscript** | |
| **Reviewer 1 comment and question** | **Responses Reviewer 1** |
|  | Dear reviewer, we are grateful for the feedback. Thank you again for the comments and suggestions, |
| This revision has substantially addressed the issues that were raised in my comments as well as those of the other reviewer. I would like to thank the authors for their work to strengthen this paper. I have only minor corrections to recommends, otherwise I approve this paper. | *Thank you dear reviewer for the feedback. We have incorporated your suggestion while revising the manuscript. |
| Authors indicate in reply to the editors that the minimal data set is in the supporting information (Response 5 to the editor). In reviewing the supporting information, I noted only the tool used to collect the data, but did not find the original data. I believe this may remain to be addressed. | Dear reviewer, we have uploaded the de-identified data set as per the inquiry. We apologize for not addressing the issue last time. |
| A comment: thank you to the authors for clarifying the reference on use with a viable fetus. Please note the Pathfinder document used as a reference is based on information from 2007, and has not been updated. It is considered out-of-date in light of several years of experience with the NASG in the field. | Dear reviewer, we truly appreciate your suggestions, for this specific inquiry we have replaced the reference with (Downing, J. et al)  DOI 10.1186/s12913-015-0694-6) although we didn’t mention it during our response last time. In addition please be informed that we have use other references together with pathfinder while developing the data collection tool including FIGO. |
| . p.12 Velcro should have an asterisk as well, as the NASG is made of neoprene and velcro. | Dear reviewer, we have corrected the sentences as per your suggestion. (**Table 3)** |
| Line 238/239 The number is 198 and percentage is 48% of respondents disagreeing with the statement according to the table. | Dear reviewer, thank you, we have made the correction. **Page 14, line 245-246** |
| Line 240. The percentage is 39%, not 61% according to the table. | Dear reviewer, thank you for your thorough review of our work. We have made the correction. **Page 14, line 248-249** |
| Line 295 "magnitude" (spelling correction) | Dear reviewer, thank you for thorough review of our work. We have made the correction. **Page 18, line 303** |
| **Reviewer 2 comment and question** | **Responses Reviewer 2**  Dear reviewer, thank you so much again for your suggestions and comments which significantly improved the manuscript. |
| There are still some concerns with the grammar. Please attend to the grammar or spelling errors in the following lines of the manuscript - 63, 67, 73, 75, 83, 95, 96 to 98 100, 105, 116, 131, 151, 159 to 160, 191, 198, 199, 207, 264, 279 and 373. | Dear reviewer, thank you. We have gone through the manuscript and made the correction as you pointed them out for us. We have made the changes on the track version of the manuscript. |
| Line 141 says the final sample size was 422 mothers?  I think this is meant to read 422 health workers. | Thank you dear reviewer, yes it health workers, we have made the correction accordingly. |
