# Supplementary material for: Utilization of non-pneumatic anti-shock garment and associated factors for postpartum hemorrhage management among obstetric care providers in public health facilities of southern Ethiopia, 2020
Source: PLoS One. 2021 Oct 28;16(10):e0258784. doi: 10.1371/journal.pone.0258784 (PMC8553034; doi:10.1371/journal.pone.0258784)
Supplement: Attachment — Submitted filename: Response to Reviewers.doc [file pone.0258784.s005.doc]

**Title:** Utilization of non-pneumatic anti-shock garment and associated factors for postpartum hemorrhage management among obstetric care providers in public health facilities of southern Ethiopia, 2020

**Authors:**

Yordanos Gizachew Yeshitila (yordanos.gizachew@yahoo.com)

Agegnehu Bante (agegnehubante@gmail.com)

Zeleke Aschalew (zelekeaschalew@gmail.com)

Bezawit Afework (bezawitafework2010@gmail.com)

Selamawit Gebeyehu ([emugebe.sg@gmail.com](mailto:emugebe.sg@gmail.com))

**Responses compiled by**: Yordanos Gizachew Yeshitila

On behalf of the authors

| **Responses to editor’s comment**  **Dear editor, Thank you for the feedback. We have addressed comments and suggestions below and within the revised manuscript (tracked changes and clean version).**  Sincerely,  Yordanos Gizachew Yeshitila  on behalf of the authors | |
| --- | --- |
| Please include the following items when submitting your revised manuscript:   - A rebuttal letter that responds to each point raised by the academic editor and reviewer(s). You should upload this letter as a separate file labeled 'Response to Reviewers'. - A marked-up copy of your manuscript that highlights changes made to the original version. You should upload this as a separate file labeled 'Revised Manuscript with Track Changes'. - An unmarked version of your revised paper without tracked changes. You should upload this as a separate file labeled 'Manuscript' | - Thank you, dear editor, we have included all the three requested items |
| **Responses to editor comments and suggestions** | |
| I thank the authors for their efforts to respond to reviewer comments. Despite this manuscript being revised twice, there remain considerable grammatical, formatting, and spelling errors that need to be addressed and corrected. Further, the non-pneumatic anti-shock garment (NASG) should be spelled out once and then the abbreviation used consistently through the remainder of the document. The manuscript would also greatly benefit from being made more concise - for example, the Introduction section states in three different places that postpartum hemorrhage is a leading cause of maternal mortality and gives the reasons for delayed care that lead to mortality. Please summarize this in one paragraph, describe the NASG in the next paragraph, discuss the introduction and use of NASG in Ethiopia briefly in a third paragraph, and then state the gap in the evidence and the reason for the paper in the last paragraph. The Results section can similarly be abbreviated by removing double-reporting of proportions in the text that are also present in the tables. Simply summarize the most remarkable finding or two findings from each table and data not portrayed in tables. Please streamline the tables by only using one decimal place. Last, please streamline the Discussion section - the results should only be contextualized with the first paragraph summarizing the main findings. The current first paragraph re-states the Introduction - please remove this paragraph and simply summarize the main findings. Please take the time to carefully revise the manuscript before re-submission and consider engaging a professional editor who is a native English speaker. | **Dear editor, we are very much gratefull for the suggestions and comments which potentially improved our work.**   - **We have made all the corrections as per your suggestion** (please kindly see the revised version with track change **)** - **We have exhaustively reviewed the manuscript and according to your recommendation, we engaged a professional editor (**the changes made and are shown the revised manuscript with track change). |
